# Psychiatry as a vocation: Moral injury, COVID-19, and the phenomenology of clinical practice

**DOI:** 10.1177/14777509231208361

**Published:** 2023-11-14

**Authors:** Matthew R Broome, Jamila Rodrigues, Rosa Ritunnano, Clara Humpston

**Affiliations:** 1 Institute for Mental Health, University of Birmingham, Birmingham, UK; 2 Embodied Cognitive Sciences Lab, 56874 Okinawa Institute of Science and Technology Graduate University, Onna, Japan; 3 Department of Psychology, 8748 University of York, UK

**Keywords:** COVID, pandemic, moral, phenomenology, burnout, Weber

## Abstract

In this article, we focus on a particular kind of emotional impact of the pandemic, namely the phenomenology of the experience of moral injury in healthcare professionals. Drawing on Weber's reflections in his lecture *Politics as a Vocation* and data from the Experiences of Social Distancing during the COVID-19 Pandemic Survey*,* we analyse responses from healthcare professionals which show the experiences of burnout, sense of frustration and impotence, and how these affect clinicians’ emotional state. We argue that this may relate to the ethical conflicts they experience when they are forced to make clinical decisions where there are no optimal outcomes, and how in turn that impacts on their own emotional state. We then further examine the notion of ‘burnout’ and the phenomenology of ‘moral injury’. Our argument is that these experiences of moral injury across a range of clinicians during the pandemic may be more prevalent and long-standing in psychiatry and mental health than in other areas of healthcare, where ethically difficult decisions and resource constraints are common outside times of crisis. Hence, in these clinical arenas, moral injury and the phenomenology of emotional changes may be independent of the pandemic. The insights gained during the pandemic may provide wider insights into the challenges of developing services and training the workforce to provide appropriate mental health care.

## Introduction

In this article, we present some ideas around the phenomenology of moral injury, and the emotional changes those who sustain such an injury may experience. Moral injury has been of increasing concern during the pandemic, particularly in clinicians who may have to make difficult ethical decisions regarding the delivery of clinical care where there are significant resource constraints (e.g. which patient might have access to an intensive care unit (ICU) bed). The argument we wish to explore is whether these experiences of moral injury of acute care clinicians in the pandemic reveal the wider phenomenology of the experience of other clinical services, in particular mental health clinicians independent of the pandemic, who often have to work in services with extreme resource constraints and where ethically difficult decisions are frequently made.

We examine Weber's phenomenology of the moral psychology of the politician, as described in his 1919 *Politics as a Vocation* lecture. In the *Talking Politics History of Ideas* podcast episode on Weber https://www.talkingpoliticspodcast.com/history-of-ideas (now published as a chapter by Runciman^1^), David Runciman outlines Weber's bleak depiction of the practice of politics, how it is inherently linked to violence, and how in turn this impacts on the individual politician. This depiction from Weber/Runciman suggests a politics that can never be pure, or wholly virtuous, and is always tied to violence. Weber's depiction sounded not dissimilar to how psychiatry is often portrayed and characterised: as a discipline and practice which may aspire to good ideals but is inherently linked to violence and coercion – and on reflection this analogy should not seem surprising. Mental health clinicians are, to some extent, political agents, and are often working for state-funded health services with duties around risk management. Their practice connects with the criminal justice system, and they have access to legal powers that enable coercive practices such as involuntary admission and treatment under mental health legislation. Hence, if Weber is correct, the harms of participating in politics may be in part shared by those working clinically in mental health services. In parallel, to this line of analysis, one of the authors (MB) was involved in developing mental health support structures for acute care clinicians during the COVID-19 pandemic at University Hospitals Birmingham, one of the largest groups of acute hospitals in Europe. In this context, the idea of moral injury and harm – a topic drawn from military and humanitarian mental health – was now being imported into clinical practice. So perhaps the topic of Weber's lecture and moral injury can mutually illuminate and unify the experience of politicians, mental health clinicians, and acute care clinicians in and perhaps also outside a pandemic.

We first briefly describe Weber's account of the harms and psychological effects of the practice of politics, drawing on his *Politics as a Vocation* lecture. Secondly, we will discuss the literature regarding the mental health problems of acute clinicians delivering care during the COVID-19 pandemic and introduce the concept of moral injury and potentially morally injurious experiences (PMIEs). In ‘The mental health of mental health clinicians outside the pandemic’ section of the article, we draw on empirical data from participants in the Experiences of Social Distancing phenomenological survey.^2^ From these accounts of poor mental health in acute clinicians, we can then begin to compare them to what we know about the mental health of mental health clinicians and begin to offer a phenomenology of the experience of moral injury, and its relation to other concepts such as burnout and compassion fatigue.

### Weber and politics as a vocation

Max Weber is a salient interlocutor to bring into a discussion on mental health care and the COVID-19 pandemic. Weber suffered from depression for a period of his life, following the death of his father, and was unable to work for several years. Further, he played a key role in the early development of phenomenological psychopathology as a friend and mentor to Karl Jaspers, where, in the *General Psychopathology*, Weber's work on ideal types was used by Jaspers to demarcate the symptoms of mental illness. Weber himself died of the Spanish Flu in 1920, shortly after delivering his famous lecture, *Politics as a Vocation*, on which we draw here.

Runciman’s^1^ discussion of Weber offers many contemporary resonances to clinical practice. Weber argues that professional politicians, and for politics to be effective, have to act in morally problematic ways, and that this comes at a cost to its practitioners. These claims are similar to those heard at some academic meetings and a significant minority of public settings where psychiatry and psychiatrists can be accused of being ‘evil’ and causing harm to those they purport to help. This possible parallel between politicians and mental health clinicians reminds us that where there are ethically difficult decisions being made, and where the workforce itself may be suffering harm, this is not just of insular interest to the members of those professions themselves but is of great importance to wider society. For politicians, this may impact on the wider general public, and for mental health clinicians, those requiring support and treatment for mental ill health. These wider groups may hence also be victims of such injury, and are likely to experience additional intersectional vulnerability and disadvantage.

Weber, in his prior work, offers a definition of the modern state, and links it to the claim that the state's function is to be able to legitimise coercion or violence. He elaborates on this further in his 1919 lecture, after Germany's defeat in the First World War:

We must say that the state is the form of human community that (successfully) lays claim to the *monopoly of legitimate physical violence* […]. The state is regarded as the sole source of the ‘right’ to use violence. Hence, what ‘politics’ means for us is to strive for a share of power or to influence the distribution of power, whether between states or between groups of people within a state (p. 33)^3^.

In developing this connection, and in his criticism of what he sees as the ‘amateur’ politicians of Imperial Germany, he argues for a professionalism of politics that recognises its inherent moral complexity:

No ethic in the world can ignore the fact that in many cases the achievement of ‘good’ ends is inseparable from the use of morally dubious or at least dangerous means and that we cannot escape the possibility or probability of evil side effects. And no ethic in the world can say when, and to what extent, the ethically good end can ‘justify’ the ethically dangerous means and its side effects (p. 8)^3^.

What is interesting, and most relevant to this article, is not only the link between political power and violence and its analogy to psychiatry, but how in the latter half of the lecture Weber speculates on the harm of practising politics to the individual. Weber believes that politics is no job for saints – there could be no utopia without the potential price of violence. One cannot be naïve to the costs of political action and hope that harm will not occur. Weber did not believe that ‘the ends justify the means’, but that the means contaminate the ends and this is what the politician needs to understand. They will have to pay this price in order to have a political life. Hence Weber's work is an element of the wider ‘dirty hands’ debate in moral and political philosophy.^4^

Weber's conclusion is that leadership of a modern state may only be possible for a very small number of people:

…a very narrow group of people who can live with the psychological strains of this double life. The double life of being a good person who is also a bad person. Of achieving noble ends through violence. Of having blood on your hands and not trying to wipe the slate clean. Weber called it doing a deal with the devil (p. 158)^1^.

Our paper takes this insight of Weber and tries to generalise it: is the political life one which clinicians in the pandemic have experienced where they have a duplex life of acting morally badly whilst trying to act well? And is this phenomenology one that mental health clinicians experience throughout their clinical practice, where, as with the state, legitimacy and coercion are intertwined? And what is the risk to those practitioners who are living this life?

### COVID-19, moral injury, and the mental health of clinical practitioners

Throughout the COVID-19 pandemic, it was almost inevitable for any individual to experience some kind of impact on their mental health. Although such an impact could lead to positive change for some, for example, those who enjoy relative isolation and lack of social contact, or a life with less commuting and commitments, for many others the pandemic has had a negative, long-lasting, and sometimes severely damaging effect on their mental health. Experience of the pandemic was further heavily influenced by class, occupation, and caring responsibilities: certain professional groups were able to work fully at home, and had access to a private external space, while others had to continue working in person (e.g. essential workers, delivery drivers, and supermarket staff), or had increased caring duties (homeschooling and absence of childcare).

Clinicians and associated medical/nursing professionals are one of the groups most seriously affected by the pandemic. Not only do they have to face significant physical threats to their health due to their frequent exposure to the infection, but they also almost constantly encounter clinical and personal situations where they must make extremely difficult, if not morally and ethically impossible, decisions. Professionally, and drawn from some of the authors’ own experience as clinicians, colleagues are often worried about the quality of care they provide, how to communicate devastating news (death and/or serious disability because of COVID-19), and treatment decisions to patients’ relatives, as well as navigating media attention and management structures within the health service, to name but a few. Further, many staff speak of a shift in their normal working pattern – an increased emphasis on ‘reactive’ care, responding to clinical events, with a rapid turnover of patients, rather than more ‘planned’ and continuous care. An ICU is usually a calm and ordered environment, but in the time of the pandemic, it became one where extra beds are created, the physical space between clinicians and patients is reduced, and where staff are wearing bulky personal protective equipment (PPE) and caring for many more patients simultaneously. Leave for staff is often cancelled and there is little flexibility about which shifts one can work, with working hours increased. Amongst these structural changes, clinicians are sometimes forced to make life-and-death decisions on behalf of the patient, for instance, how to triage care, who is able to access an ICU bed, and when to terminate life support. This is coupled with a change in the support clinicians are able to offer to patients’ loved ones who may experience an understandable (but nonetheless traumatising) response to the death or serious illness of their family member, support that is further complicated by frequently having to deliver the devastating news remotely. A distressing UK example of this, early in the pandemic, is when a 13-year-old boy died alone in a hospital after testing positive for COVID-19, where his family was prevented from visiting https://www.independent.co.uk/news/uk/home-news/coronavirus-boy-death-hospital-london-youngest-uk-ismail-abdulwahab-a9439526.html.

On a more personal level, clinicians rarely, if ever, fall into a group where they can enjoy relative isolation away from social contact. Many clinicians have personal caring responsibilities irrespective of their long shifts and others have relatives and loved ones overseas who they are unable to visit due to travel restrictions. They have often lost childcare support from their extended family or are no longer able to see their children's grandparents. To put simply, clinicians are also human beings who lead independent lives outside the hospital/professional settings, and this is one of the key aspects neglected by media reports of ‘NHS Heroes’, for example. As well-intentioned as many of these campaigns are for National Health Service (NHS) staff, some of them could, in fact, have a dehumanising and demoralising effect on healthcare personnel, especially when many are working flat-out with limited resources, poor working conditions, and salary, and on occasion even less empathy and understanding from the public. Sometimes the very expectation of sustaining the role of a ‘hero’ is difficult enough internally for the clinician; when confronted with (even if simply perceived) failures in maintaining this role, these expectations may often lead to very real consequences and, in extreme cases, tragic events such as clinician suicide.^5^

Given these experiences, the concepts of moral injury and moral distress have been utilised to try and understand the impact of working through the pandemic on clinicians. These concepts are originally derived from military mental health, being used to understand the impact of experiences of conflict on armed forces personnel, but they also included insights from the experiences of humanitarian aid workers and journalists, who may be present in areas of conflict or disaster. Moral distress is a psychological harm that arises when people are forced to make, or witness, decisions or actions that contradict their core moral values. If this distress is sustained, moral injury can develop which is linked to PTSD-like experiences and burnout. Moral injury results from the individual facing overwhelming demands for which one feels unprepared and where actions or inactions challenge an ethical code. A related term, Potentially Morally Injurious Experiences (PMIEs), is used to describe the experiences which may lead to moral distress or injury and includes experiences linked to ‘perpetrating, failing to prevent, bearing witness to, or learning about acts that transgress deeply held moral beliefs and expectations’.^6^ Moral distress/injury is the strong emotional and cognitive response that occurs following these experiences and events, and frequently includes disgust, anger, guilt, and shame. Recent critiques have also highlighted how such notions of moral injury should be broadened to consider the intersubjectivity of the experience, how such experiences sit within a wider moral ecology, and how thinking beyond the individual, highlights issues of structural violence, and the ultimate impact on those most vulnerable.^7,8^

Fortunately, suicide amongst clinicians as a consequence of COVID-related demands is not common, although the true number of suicides as a direct result of the pandemic in healthcare professionals could well be underestimated.^9,10^ For most clinical practitioners, negative impacts on their mental health manifest as symptoms of depression, anxiety, post-traumatic stress disorder (PTSD), alcohol/substance misuse, and less commonly perhaps, quasi-psychotic or psychotic-like experiences (e.g. transient hallucinations and paranoid thoughts). It is apparent that some of these symptoms are more widely recognised than others, with more psychotic-like experiences causing even greater anxiety in the clinician due to their fears around the response from occupational health services, their professional organisation, and concerns around fitness to practice.

These latter worries undoubtedly add to the burdens faced by the affected clinician and can exacerbate their worries about their mental health as the negative consequences from human resources departments in their hospitals can frequently directly impact their livelihood in an economic climate that is already very difficult, or lead to concerns about their professional practice and registration. As such, clinicians are often reluctant to seek help for themselves until they reach a crisis point, where their fitness to practice might be affected in very real terms. This is, therefore, an irreconcilable dilemma for the clinician regardless of the pandemic, however, given the serious limitations on resources and personnel (e.g. colleagues off sick due to COVID or COVID-related health conditions, and leave being cancelled), clinicians may be even more reluctant to ask for help at times when it is most needed.

Although systematic investigations are still lacking, emerging empirical evidence from both qualitative and quantitative studies indicate that unsurprisingly, just like their patients and the general public, clinicians are heavily impacted by the pandemic's negative effects on their mental health.^11^ Early data suggest a four-fold increase in mental health problems in UK clinicians, with 30% experiencing severe anxiety and/or depression.^12^ In this study, those who were female, who worked in the more clinically acute areas of the service, had increased workload, and had concerns over PPE availability, were most at risk of these negative outcomes whereas those clinicians who had access to opportunities to share their experience of stress with peers, and had supervisory support in their ethical decision-making, were likely to have fewer symptoms. There are wider adverse outcomes for clinicians including relationship and family breakdown, career choice with individuals leaving healthcare altogether, or moving into less acute or non-state-funded healthcare provision, as well as the development of less helpful coping mechanisms (e.g. alcohol or drug misuse). There is the wider issue too of ‘presenteeism’ – where staff may be unwilling or unable to absent themselves from work due to their poor mental health, but are functioning sub-optimally and where poor, slow, or incorrect clinical decisions may have negative consequences for patient care. Interestingly, the needs of clinicians seem to be sometimes at odds with those recommended by various support and well-being guidelines at least in the UK.^13^ For example, whereas most guidelines focus on individual resilience training, clinicians place more emphasis on the available support structures in place at work and in the wider healthcare professional community. Further, clinicians are concerned that individual support interventions are not always based on the lived experiences and working structures of those who suffer from poor mental health, especially when clinicians are almost actively prevented from partaking in these interventions due to understaffing, burnout or severely over-committed schedules that were already prohibitive even at times before the pandemic.

In response to the urgent demand for effective mental health support for clinicians and other frontline healthcare professionals, a group based in New York City^14^ trialled the implementation of a ‘mental health PPE’ programme where both psychiatric liaison teams and crisis resolution teams are embedded in accident and emergency departments and dedicated COVID-19 hospital units, with telephone hotlines available for staff to call 24/7. The group reported a very positive reception and take-up by both frontline staff and hospital leadership teams, with recommendations to continue the programme even beyond the pandemic. A remaining concern, however, is the question of ‘who heals the healers?’ What happens when psychiatric teams are put in place for helping frontline (e.g. emergency or intensive care) staff experience burnout and mental health symptoms of their own kind? It seems that when faced with reactions to trauma, grief, and loss on a mass scale, there is no single solution that will accommodate everyone's needs in an ethically and morally sound manner; and this may be a mechanism by which moral injury is perpetuated in clinicians, for whom vulnerability and failure are not only stigmatised but also directly play into the vicious circle of guilt, isolation, and responsibility. What does seem to be the case is that ensuring rapid access to mental health care, avoidance of debriefing, ensuring access to basic needs (food, fluids, and rest), and maintaining work and personal-social relationships are likely key. Traditional exposure-based and trauma-focused therapies may not be effective, whereas approaches that focus on self-forgiveness, acceptance, self-compassion, and (if possible) making amends, might hold more promise.^15,16^

### Socio-political and emotional experiences of the pandemic among clinicians

To complement the brief review of the literature above, we will now summarise the work of an international group studying the experience of the pandemic as lived from a phenomenological perspective, a group to which the authors (MB, CH, and JR) belong and is summarised by Froese *et al*.^2^ We have used this sample to extract data on clinicians’ experiences of the pandemic, across the international sites. This mixed sample of clinicians allows us to try and distil the experience of moral injury in those providing healthcare during the pandemic. We did not ask specific questions about moral injury or distress, yet content relating to these experiences occurred in the participants’ feedback. From these experiences within the pandemic, we wish to argue that some of these features occur more universally in mental health clinicians independent of the pandemic.

## Methods

People's lived experience of the pandemic was investigated using an open-ended SurveyMonkey anonymous survey ‘Experiences of Social Distancing during the Covid-19 Pandemic’ conducted in 2020 (5 June to 31 July 2020) and in 2021 (7 April to 31 July 2021).^a^ The first round (2020) was distributed through press releases from the academic institutions involved, publications, presentations, Facebook advertising campaigns, and the authors’ social networks. In the second round (2022), participants were recruited by email among the 1036 people who agreed in the previous survey to take part in a follow-up study. The survey consisted of six sections with a total of 42 questions (see Froese *et al*.^2^), and participants were free to answer as many questions as they wanted.

This article discusses the results obtained from a secondary analysis of UK participants working in healthcare. This was conducted to gain insight into, and a better understanding of, narratives of moralities, moral injury, and life both at, and outside of, work amidst healthcare practitioners on the front line of the COVID-19 pandemic in the UK. It does not seek conclusive results, but rather aims to contribute to existing investigations on moral injury and healthcare practice during the COVID-19 pandemic.

All questionnaire responses were imported verbatim into qualitative data analysis computer software ATLAS.ti and NVivo by two independent coders (JR and RR). This software was used to organise the data and to analyse them in a systematic yet flexible and creative way. Our focus was on surveying UK participants who reported working in hospital and healthcare environments and their lived experience of the pandemic. JR manually went through the ‘occupation section’ to check if any of the healthcare participants were missing. Secondly, JR compiled the files and shared them with all team members for comparative analysis. JR and RR read the survey questionnaires in their entirety to understand participants’ general experience of the pandemic.

JR and RR read through all the responses to questionnaire questions and separated the lived experience of healthcare practitioners during the pandemic according to contexts. JR and RR selected a series of answers that healthcare participants provided related to the overall lived experience of the pandemic and what feelings people may report having (e.g. guilt, anger, disgust, sadness but also positive emotions). JR and RR separated ethical decisions and moral narratives related to their working environment, safety, social distancing, dealing with stress, outside the work environment, family and friends, communication, coping, and others.

JR and RR chose a reflexive thematic analysis method approach because it allows for theoretical flexibility in the analysis of qualitative data.^17^ The study adhered to the six-phase analytical process that Braun and Clarke propose: (1) the researcher becomes familiar with the data, (2) generates codes, (3) constructs themes, (4) reviews potential themes, (5) defines and names themes, and (6) produces a report.^17^ All members met regularly to discuss and review codes, the divergent interpretation of data, and reach a consensus on inductive codes. As the coding scheme was refined, all members contributed to the development of the final themes and sub-themes (Table 1). There may be some overlap between themes and sub-themes, and hence, they may not be wholly distinct from one another. The analysis, coding process, and results were discussed with MB and CH to improve reliability and minimise bias. MB selected illustrative quotes for this article.

**Table 1.** Overview of themes and sub-themes generated through reflexive thematic analysis.

Themes	Sub-themes	Narratives quotations
Hospital/Healthcare environment: The Politics of the Workplace	Safety at work Burnout Connection and interaction with patients PPE	I have been seeing 8 clients a day 5 days a week for psychotherapy. This is more online contact than I’ve ever had personally or professionally before. I am exhausted by it and struggle to find the energy to be fully present with my children in our home. […] I think being exhausted from working online is a reason for not having more contact with family or friends (**EN_00_1434 clinical psychologist)** At work for a long time, it was difficult to think about anything except COVID-19. I work in neonatal intensive care which has not had covid cases, but it seemed to affect everything we did. And wearing masks for 13-h shifts is really challenging and makes it difficult to concentrate and to communicate (**EN_UK_2261 nurse)** I find it much harder to deal with non-face-to-face communication. I rely on physical cues to determine when people are being authentic, and especially to tell when people are being sarcastic or not. So, it is not so much my ability to trust other people as my ability to trust my interpretation of what they are meaning (**EN_UK_1072 psychologist)**
Coping with the pandemic: Reciprocal relations between the individual and their socio-political worlds	Positive coping (i.e. walks in nature; exercise; outdoor activities) Maladaptive Coping (i.e. turning to alcohol)	Take good care of my health and well-being so I can be of help to others: meditate, seek spiritual guidance, laugh, hug my neighbor's dog, adopt a cat soon, eat good food, be outdoors a lot, keep in touch with friends (**EN_00_1594 psychiatrist)** I’ve missed socializing with friends. I’ve talked to them on the phone. We’ve also drank more alcohol at home than usual. Not healthy but it's helped us feel more normal (**EN_UK_0046 nurse)** My usual coping mechanism is alcohol, but I know this doesn't work. I initially started drinking more but have now cut back as it did not help. I struggle to finding positive coping strategies. My usual method for distraction is going on the internet, watching TV, or Netflix (**EN_UK_0924 nurse)**
Felt Moralities	Personal morals regarding social distance, isolation, safeguarding others, and self-protection Societal moral awareness: mistrust others, responsibilities for others, protecting Mistrust, disappointment	I take personal responsibility for my actions. Others, I don't always trust to be responsible (**EN_UK_ 0866 nurse)** I do, however, have significantly less trust in the government due to the response to the pandemic due to the Dominic Cummings debacle (**EN_UK_ 0046 nurse)**
Shifts in worldview	Family values/interpersonal relationships Quality time and enjoyment Appreciation of nature Ambiguity, uncertainty, and emotional distress Uncertainty about the future	I value more each day with my family. Because I remember that can be the last. That made me realize the blessing it is seeing them again the other day. I feel calmer in some way that I can read more, sing more, and stay alone more without time spent in everyday overwhelming traffic and crowds (**EN_UK_1493 physician)** The fact I can die at any time and the challenges my family will have to face in case that happens. That scared me. It still scares me (**EN_00_1493 doctor)** Yes, increased my sense of my own mortality, my fear of getting ill; my fear of what will happen to the economy and my fear of a dystopian future. Especially as, at the same time, we have also seen a rise of far-right/ left parties, intolerance, nationalism, and breakdown of the rule of law/democracy in the world (**EN_UK_ 2003 psychotherapist)**

### Findings: Moral distress in the pandemic and beyond: A phenomenological cross-analysis

The argument we wish to make in this article is that the PMIEs and resultant moral distress and injury that have been reported in acute care clinicians during the pandemic, and are reflected in the themes and quotes illustrated in Table 1, are common in mental health clinicians at other times independent of the pandemic. This section therefore begins with a descriptive account of the themes identified in the responses to the survey. The themes are then developed further through a cross-analysis of common phenomenological features emerging from the literature on the mental health of mental health clinicians outside of the pandemic. This allows us to start to sketch a more global account of the phenomenology of moral injury beyond the experience of the pandemic.

The themes described refer to moral, political, emotional, and interpersonal experiences related to the pandemic experienced both at work and outside the workplace by clinicians. While survey respondents in this study were not necessarily working within the mental healthcare system, our analysis suggests that common phenomenological features of moral distress can be identified across different caring professions and points to a much wider-ranging phenomenon.

### Theme 1. Hospital/healthcare environment: The politics of the workplace

For some participants, the work environment had drastically changed during the pandemic. Whether working from home or in a clinic or hospital, participants reported long hours of work, difficulty communicating with patients, the need for additional safety equipment, and overall fatigue. Burnout appears to have physical and mental health implications. For example, some described constant concern about death rates of COVID-19, and feelings of impotence or frustration towards the situation. Yet, others also reported less preoccupation with safety equipment and the risk of infection because of the high-risk nature of their job. A few examples are provided below.

EN_00_1434 clinical psychologist
  I have been seeing 8 clients a day 5 days a week for psychotherapy. This is more online contact than I’ve ever had personally or professionally before. I am exhausted by it and struggle to find the energy to be fully present with my children in our home. […] I think being exhausted from working online is a reason for not having more contact with family or friends.
EN_UK_2261 nurse
  At work for a long time, it was difficult to think about anything except covid. I work in neonatal intensive care which has not had covid cases, but it seemed to affect everything we did. And wearing masks for 13-hour shifts is really challenging and makes it difficult to concentrate and to communicate.
EN_UK_1072 psychologist
  I find it much harder to deal with non-face to face communication. I rely on physical cues to determine when people are being authentic, and especially to tell when people are being sarcastic or not. So, it is not so much my ability to trust other people as my ability to trust my interpretation of what they are meaning.
EN_00_1515 surgeon
  I have had some fleeting feelings of despair and have cried alone, especially when seeing firsthand the CCU [critical care unit] in hospitals and clinics overwhelmed with patients young and old, the many faces of the Covid disease and the overall feeling if impotence with my colleagues that this disease leaves us.
EN_00_1578 nurse
  Do not put a hand on the shoulder of a loved one whose mother / father is dying. I find it unnatural. Doesn't feel right…
EN_UK_2680 theatre nurse
  My job is an essential job, so I have security there. As for getting infected, as a nurse in the NHS for 40 years, I've been exposed to every bug going so to me it's a day in the office! Also because being a theatre nurse wearing masks is like putting on me knickers! Doing something I don't even think about. Wearing PPE is part of my role anyway. I just do it.
EN_Mx_ 0131 nurse
  I feel physically low on energy and tired. I feel anxiety and depression*.*

Parallels can be drawn intuitively between the politics of the workplace as described by the survey participants in the pandemic, and as they are lived through by mental healthcare professionals in their daily work environment. Research in the field seems to converge on similar findings, often involving job characteristics on the one hand (e.g. workload, work relationships, and work environment), and personal characteristics on the other (e.g. age, sex, years of experience, and personality traits). For instance, research investigating well-being and burnout among mental healthcare professionals has previously pointed to the role of work-related factors. Two recent systematic reviews of the literature on poor resilience in psychiatrists^18^ and burnout in applied psychologists^19^ found a strong and consistent relationship between burnout and excessive workload, including working long hours and high work demands. Gerada^20^ discusses factors including the experience of patient violence and suicide, and the impact of any resultant blame; the limited resources for treatment and crowded and inadequate inpatient units; the culture of mental health units; the high work demands and the responsibility for patients often without the authority to change care; and the experience of conflict between duty to employers vs. duty to patients. These characteristics map onto those likely to cause PMIEs.

Interestingly, in these papers, the authors highlight not only the role of an objective increase in the number of hours or patients, but also the negative impact of time spent on the perceived *wrong* type of work (e.g. administrative work). This not only led to increased emotional exhaustion but also to a decreased sense of accomplishment.^21^ While the example of excessive administrative workload may seem banal, as compared to more troublesome ethical dilemmas such as those concerned with involuntarily and coercively treating someone, potentially similar and subtle moral processes are at work in both cases. Such moral processes and influences appear to be the dominant features of the current unappealing state of affairs in mental healthcare. Armstrong^22^ refers to such states of affairs using notions of bureaucratic accountability, risk, and clinical autonomy. As 21st-century mental health services are reconfigured as businesses that are ‘accountable’ (driven by market mechanisms), the role of mental health clinicians is similarly reconfigured away from that of an ‘expert’ professional making autonomous clinical decisions, and arguably closer to their core ethical and professional values, to something more akin to a ‘bureaucrat’ whose action and judgment appear ethically suspect and are under constant scrutiny. In this situation, psychiatrists can either ‘learn the art of self-delusion, convincing ourselves we are not letting patients down but, instead, doing the clinically appropriate thing’ as Beale^23^ puts it, or are destined to find themselves drained of compassion and emotionally exhausted by an excessively risk-averse and under-resourced system.

### Theme 2. Coping with the pandemic: Reciprocal relations between the individual and their socio-political worlds

When asked how they coped with the changes brought about by the pandemic, some survey participants mentioned adopting a positive attitude and enacting helpful strategies towards maintaining their well-being. For example, some sought meditation and spiritual guidance, a closer relationship to the natural world, or turned to interpersonal relationships and the use of digital media to communicate and strengthen their relationship with others. In contrast, others found it hard to maintain a positive attitude during the pandemic. A few of the participants shared feelings of anxiety, depression, turning to alcohol, and disappointment towards friends.

These responses offer some insights into the phenomenology of reciprocal relations between the self-identity of healthcare workers and the collective socio-political communities during the pandemic. They provide some evidence of the complexity of collective moral and emotional changes experienced during the pandemic: issues of anger and distrust, increasing concerns about mortality and the vagaries of time and existence, exhaustion and burnout, differences in the experiences of being with others, and ranges of coping mechanisms.

While the reciprocal relations at work between the self and the collective, and their (positive and negative) effects were made particularly salient by the pandemic itself, the same themes can be identified as an invariant feature of the experience of moral distress in mental healthcare workers. Indeed, we understand providing care to those with complex mental health needs can be stressful for the individual to say the least. High levels of stress, job dissatisfaction, burnout (emotional exhaustion, depersonalisation, diminished sense of personal accomplishment), poor well-being, higher rates of psychiatric symptoms, and suicidal ideation are commonly observed among psychiatrists^18,20^ and other mental healthcare professionals.^24^

More generally, moral distress is a type of psychological harm arising when people are forced to make, or witness, decisions or actions that contradict their core moral values. Psychiatric practice presents clinicians daily with situations in which they feel that they may fail their patients: denying adequate care to help-seeking individuals because of the chronic lack of hospital beds and community resources; inability to access psychotherapeutic interventions; enacting forms of coercion under the Mental Health Act whilst perpetually questioning whether an infringement to a fundamental right to liberty can be justified under such premises; prioritising patients’ access to services on the basis of ‘severity’ criteria whilst being fully aware that when such criteria are met, it is often too late. While this is not a comprehensive list, many UK psychiatrists (and other mental health clinicians internationally) are likely to recognise such experiences as a significant part of their day-to-day job, with ensuing high levels of emotional exhaustion and burnout.

EN_00_1594 psychiatrist
  Take good care of my health and well-being so I can be help to others: meditate, seek spiritual guidance, laugh, hug my neighbour's dog, adopt a cat soon, eat good food, be outdoors a lot, keep in touch with friends.
EN_00_1157 carer
  I have to be my own support system. I am naturally optimistic and energetic. I was erratic abut meditating and I have now made that a mandatory start to my day. I listen to music. I love reading so I read when I can. I love walking in nature and am very blessed to be in a location with miles and miles of walks.
EN_UK_0046 nurse
  I’ve missed socialising with friends. I’ve talked to them in the phone. We’ve also drank more alcohol at home than usual. Not healthy but it's helped us feel more normal.
EN_UK_0924 nurse
  My usual coping mechanism is alcohol, but I know this doesn't work. I initially started drinking more but have now cut back as it did not help. I struggle to finding positive coping strategies. My usual method for distraction is going on the internet, watching TV or Netflix.

### Theme 3. Felt moralities: Moral injury as involving political emotions

During the pandemic, survey responses were suggestive of the role of political emotions^25^ as an essential dimension of the pandemic experience. Participants described wide-ranging ethical sentiments or ‘moralities’ (value-laden, experiential layers that constitute the lived reality of healthcare practitioners) that seem to be at stake during the pandemic. Healthcare practitioners’ ideas about safety and social distancing issues are strongly associated with feelings of responsibility and moral obligation related to government guidelines. Many participants reported worrying about the safety of their families and loved ones and acting to reduce the risk of transmission, while feeling angry at those who were not following the guidelines. Others expressed shock and distrust towards the government, and their peers’ or strangers’ behaviour. For instance, to keep family members safe, a doctor may isolate himself/herself in a hotel. Quotations are provided below.

As Szanto and Slaby^25^ put it in their compelling account of political emotions, ‘the political – the realm in which we negotiate our plurality and differences with a view to freedom, power, individual autonomy, collective recognition or our forms of living-together – is essentially affective. […] The political is affective because it fundamentally deals with what matters to us, what we value, fear or desire, or what concerns us – us as a polity’. The survey responses highlight how people's emotions during the pandemic were overwhelmingly modulated by their shared or conflicting values (with other workers, or members of the public), which were, in part, politically shaped, so that a constant negotiation was at play between emotions, judgment, and action – at times resulting in moral distress and injury.

Another parallel can be drawn here between the political emotions that were made salient during the pandemic and those that are lived through by mental healthcare clinicians in their day-to-day profession. In this latter case, the normative infrastructure that governs the political life of mental health professionals will necessarily feed into the individual's *felt experience*. In Weberian terms, this may lead to those ‘psychological strains’, as described above, resulting from the double life of being a good person who is also a bad person.

In and out of the pandemic, the political closely intersects and interacts with the emotional. Concurrently, the shared normative framework to which professionals must respond to, seems to significantly modulate individuals’ emotional experiences. In a situation of chronic job demands and stress, mental health clinicians not only have to be loyal and respond to their institutions, but they also have to listen and respond to the pain of fellow human beings under their care. Ultimately, this may give rise to a clinically significant constellation of negative emotions and psychological distress. Within the psychology literature, this is commonly cashed out in syndromic terms when referring to the psychiatrist (in this case, turned patient) as *being affected* by ‘burnout’ (BO) or by ‘compassion fatigue’ (CF).

UK_En_ 1493 physician
  I was working many days in a week in the frontline, decided to isolate even from my family nucleus. I went to a hotel. But then I changed my place and shifts in the hospital and decided to get back home. That made me feel better.
UK_EN_1512 physician
  I feel angry with those who do not follow the rules. I have felt upset that I had to go to work when others got to stay home and worry that the economic impact for the country and my kids will be huge.
EN_00_ 1594 psychiatrist
  I am aware of how many people ignore the guidelines. I meet my clients on video platforms, so I both see and hear from them how they are not at all social distancing. That is why my office remains closed even when I could open it legally with protections in place.
EN_UK_ 0866 nurse
  I take personal responsibility for my actions. Others, I don't always trust to be responsible.
EN_UK_ 0046 nurse
  I do, however, have significantly less trust in the government due to the response to the pandemic due the Dominic Cummings debacle.
EN_00_1578 nurse
  I can get very sad about the whole situation. The WHO destroys humanity, they keep hammering away. They have to counteract super spreader events !! Micro drops instead of the big drops take care of the seriously ill. Ventilate!

### Theme 4. Shifts in worldview

Some healthcare practitioners reported that their experience of the pandemic had heightened their awareness of, or reflection upon, mortality. Participants may be concerned about their death and the consequences of this for their families, whereas others may be concerned about the pain of death. When asked whether anything in their lives has changed in the ‘outside world’, participants’ answers included both positive and negative aspects. For example, some felt a sense of increased unpredictability and uncertainty about the future as well as doubting oneself more, while others reported feeling calmer, appreciating nature, and having more time to connect with oneself and with loved ones.

EN_UK_1493 physician
  I value more each day with my family. Because I remember that can be the last. That made me realize the blessing it is seeing them again the other day. I feel calmer in some way that I can read more, sing more, and stay alone more without time spent in everyday overwhelming traffic and crowds.
EN_00_1493 doctor
  The fact I can die at any time and the challenges my family will have to face in case that happens. That scared me. It still scares me.
EN_UK_ 2003 psychotherapist
  Yes, increased my sense of my own mortality, my fear of getting ill; my fear of what will happen to the economy and fear of a dystopian future. Especially as, at the same time, we have also seen a rise of far right/ left parties, intolerance, nationalism, and breakdown of the rule of law/democracy in the world.
EN_UK_ 2261 nurse
  I have felt much more anxious. A pervasive, non-specific anxiety about the uncertainty. It feels sometimes like I am living in a dreamworld. But I am not sure if that dream world was life before Covid, or life now.
EN_00_14341 psychiatrist
  I have noticed that I am doubting myself more (in every area but not in my professional life) and doubting my ability as a mother more, as I feel guilty about having less time for my children, and about the amount of screen time they have had during this time. I have also noticed older anxiety driven behaviors returning, such as being overly agreeable and not asserting myself.
EN_UK_00_1680 nurse
  Nature has been amazing this year! There's butterﬂies I haven't seen, wild ﬂowers everywhere, the air seems clearer – sadly human nature doesn't appear to have learned the lesson. The 6 weeks of heavy lock down was amazing – no litter, no traffic – now it's back to normal.
EN_00_1961 nurse
  Have more free time. I now appreciate how busy my life was before Covid-19 and welcome some down time. Not so much positive changes as the fact that strong relationships that existed are still intact. In myself, I realize now that I was living a frantic, busy life. I am now enjoying time alone and doing things alone and having time to do some things that never got done–organized closet; getting pictures organized, reaching out to more family members.

We suggest that these responses do offer some insights into the phenomenology of healthcare workers during the pandemic and provide some evidence to the complexity of affective and moral change experienced during the pandemic: issues of anger and distrust, increasing concerns about mortality and the vagaries of time and existence, exhaustion and burnout, differences in the experiences of being with others, and the range of coping mechanisms. Our empirical data, particularly those from themes 1 (hospital/healthcare environment) and 3 (felt moralities), and from respondents EN_00_1515, EN_00_1578, UK_EN_ 1493, and UK_EN_ 086, demonstrate how ethical and emotional experiences intersect with professional actions and wider choices around personal and family life. Our contention is such emotional and ethical harms may themselves lead to distress, worsening of mental health, and in some, the onset of psychiatric illness, particularly depression, anxiety, post-traumatic stress disorder, or alcohol/drug misuse.

But there is something more to be added to the psychological ‘syndromic’ articulation of these experiences, which remains largely invisible, as psychiatrists:

…use themselves as the tools of their trade, as they listen, interpret and reflect back to their patients. The courageous actions of psychiatrists are largely invisible. It takes enormous mental strength to remain focused when having to listen to the psychic pain of patients […]. This intense doctor-patient relationship is difficult and unpredictable. Patients evoke emotions such as the need to rescue them, and a sense of failure and frustration when the illness does not respond to treatment (p. 217).^20^

## The mental health of mental health clinicians outside the pandemic

The argument we wish to make in this article is that the difficult ethical choices being made, and the resultant moral distress and injury that have been reported in clinicians during the pandemic, are common in mental health clinicians at other times independent of the pandemic, are hence more universal and ubiquitous in this specific clinical arena. We would also suggest that there may be other areas of practice where this is likely the case: for example, children's social work. Indeed, while the COVID-19 pandemic has brought attention to experiences of moral injury in front-line key workers, including mental healthcare staff, the latter group of professionals is certainly not new to such experiences. Rather, some may describe experiences of moral injury to be endemic to the mental health profession,^23^ and we have detailed some of the empirical evidence of mental health issues in mental health clinicians above.

To remind ourselves, moral distress is a type of psychological harm arising when people are forced to make, or witness, decisions or actions that contradict their core moral values. Mental health practice is full of such potential for moral distress and is often accompanied by resultant exhaustion and burnout. What gets lost in this framework is the figure of the psychiatrist, or mental health clinician, as a human being caring for another human being and confronted with (often impossible) moral decisions and emotional distress on a daily basis. As we have seen, this may be due to different factors including resource deficits, responsibility without due authority, conflicts between duty to employers and duty to patients, high work demands, inadequate and crowded inpatient facilities, and the inability to provide the care that one believes one's patients need, be it access to a calm and safe inpatient unit, to skilled psychotherapy, or for physical healthcare.^18^

Ultimately, this ‘double life’ that many clinicians find themselves living may give rise to a clinically significant constellation of negative emotions and psychological distress. Within the psychology literature, this is commonly cashed out in syndromic terms when referring to the psychiatrist (in this case, turned patient) as *being affected* by ‘burnout’ (BO) or by ‘compassion fatigue’ (CF). While the meanings and usage of these terms vary widely across a largely uncharted cross-disciplinary territory, the psychological literature has attempted to clarify the constructs of BO and CF by providing operational definitions. While little attention has been given to the differing phenomenology of these experiences, such definitions do differ in some key respects.

First introduced in the scientific literature by American psychologist Herbert J Freudenberger in 1974,^26^ BO was subsequently defined as ‘a prolonged response to chronic emotional and interpersonal stressors on the job’ (p. 397).^27^ This encompasses three distinct dimensions: (a) emotional exhaustion, referring to the loss of physical and emotional resources and lack of energy; (b) depersonalization/cynicism, referring to the negative attitudes (e.g. becoming distant and uncaring) developed by the person towards their clients, peers, and organisation; (c) a diminished sense of personal accomplishment. Although other definitions have been proposed, theorists seem to agree on the fact that BO largely derives from long-term occupational stress and has emotional exhaustion as a core component.^27–29^ Different from CF, BO does not result directly from working with traumatised clients and from exposure to traumatic material or events. Rather, it appears to be more typically related to workplace factors such as high demands and lack of resources. CF, in contrast, has been described as the ‘natural consequent behaviours and emotions resulting from knowing about a traumatising event experienced by a significant other – the stress resulting from helping, or wanting to help, a traumatised or suffering person’ (p. 7).^30^ Therefore, while the two constructs seem to differ quite clearly on the basis of the specific cause of the stress (be it organisational resources in the case of BO, or some forms of client contact in CF), their differential phenomenology is, however, still unclear. Even less clear is the relationship between these constructs and the experience of moral injury.

Moral injury is not usually conceived of as a ‘syndrome’ or a ‘disorder’. Rather, in psychiatry, it is currently considered to be a risk factor for mental disorders, especially complex PTSD, depression, and substance misuse amongst others.^31^ From our own empirical data above, and review of the mental health of mental health clinicians, some phenomenological themes do emerge. Concerns about mortality become heightened, with increased uncertainty about the future, and more complex and conflicting emotional ties. Responsibility towards others alters intersubjectivity, and, for many clinicians, there is a great sense of loss of what they were, their internalised conception of their professional role, and their sense of connection and belonging in healthcare and wider society. These phenomenological changes resonate with recent work detailing experiences of trauma and grief.^32–34^ Experiences of trauma can lead to both a sense of a foreshortened future, as well as a lack of certainty and trust in such a future,^32^ something we see in our data and review of clinicians’ experiences. Wilde,^34^ drawing on Walther's conception of ‘habitual unification’ argues that trauma offers a challenge to one's default sense of belonging, and in our data, this change in intersubjectivity and connection to others was clear and in relation both to professional role but also to their connections with the wider society. From speaking to many clinicians, there is also a profound sense of loss following the pandemic – grief not only for the loss of people and as bereavement, but also a loss of professionals’ own sense of themselves and their careers, their ‘life-project’ going forward, and a change that leads to a re-evaluation of their future. This, alongside health, pay, and conditions, may be an important driver to the current workforce challenges in the NHS.

As we learn more from the pandemic experience, further work is needed to conceptualise moral injury. To this end, phenomenological investigations into the what-it-is-likeness of moral injury and moral distress may be a promising point of departure. Yet, there have been relatively few explorations and direct comparisons of the phenomenology of different kinds of moral distress – particularly through the lens of BO and CF.^35–39^ This small body of literature, mostly using phenomenological-interpretive, hermeneutic, or existential qualitative methodologies, helps to illuminate some of the meanings of *being and becoming* burnout or fatigued. Across cases of BO and CF, and irrespective of the cause of the reported stress, a strong feeling of being too ‘emotionally tormented’ emerges as a core phenomenological feature. Narratives by different healthcare professionals appear dominated by themes of ‘being torn between what one wants to manage and what one manages’,^36^ ‘I should be able to accomplish’,^35^ and the ‘professional face’ – as described by a student nurse in a study of compassion fatigue using poetry as a source of data^37^:

‘Professional face!’ though I feel defeated

I keep wearing the professional face

The overly interested tone of voice

Keep listening and listening and listening.

Thirteen hours and forty minutes later

I’m not sure if my feet are still at the end of my legs I’m not sure I have legs

[…]’

## Conclusion: Towards a phenomenological account of moral injury in medicine and psychiatry

The cross-analysis we have offered can help us to sketch an initial account of the phenomenology of moral injury, in and outside of the pandemic. This account allows us to translate Weber's intuitions on the practice of politics into the life of clinicians who, especially when working in mental health services, seem to experience a duplex life of acting morally badly whilst trying to do good. In the context of clinical practice, as with political life, where legitimacy and coercion are twined together, the political becomes overwhelmingly affective as individuals’ emotional experiences are modulated by reciprocal interactions with collective values. In this sense, the experience of moral injury appears to involve profoundly political emotions emerging from reciprocal and dynamic relations between the individual, the socio-political communities to which they belong, and the patients towards whom they have a duty of care. On the part of the clinician, this entails a process of constant (re)negotiation between emotions, judgments, and actions, which can sometimes lead to clinically significant levels of emotional distress – what in psychology is variously referred to as burnout or compassion fatigue.

Weber's lecture, *Politics as a Vocation,* was a spur to the writing of this paper, as it does a proto-phenomenology of occupational moral injury where the ethical harms to an individual are seen in the light of the choices one has to make in carrying out their work. As such, Weber's account follows on from similar philosophical worries of the impact of work and industrial society on the individual as detailed in Rousseau, German Romanticism, Marx, and the American Transcendentalists, amongst others. However, where Weber's lecture is more relevant is his focus on there being a lack of optimal choices and the impact on making these choices has for a politician. Given these sub-optimal ethical choices are not wholly the domain of politicians (or rather, other professionals share some structural similarities with politicians, for example, the power of coercion, managing scarce resources, and working with the criminal justice system), we believe Weber's argument can be extended to other professions and vocations.

In this analysis, our main conclusion is that medicine and healthcare can be seen as a kind of politics, in a Weberian sense, with the moral injury suffered by clinicians as an inevitable consequence of the non-optimal decisions that they can be forced to make, and this is amplified in times of increased clinical need and insufficient resources (e.g. in times of military action or a pandemic). We have demonstrated some of these phenomenological changes by utilising quotes from healthcare practitioners during the pandemic. Our second conclusion is that due to the lack of resources for mental health care, and the lack of consistency in care for those with mental ill-health contributing to unequal outcomes, this link between clinical practice and moral harm that has become emphasised in the pandemic is more of the norm than the exception for mental health clinicians in their work, both during and outside of the pandemic. We have demonstrated this by showing how each of the themes generated by the survey analysis has a clear parallel with the experiences of moral distress reported in the literature on the mental health of mental health clinicians. As with professional politicians, Weber would counsel mental health clinicians to think about the challenges this work can bring and how to manage the psychological and emotional complexities of practice, and for organisations to have a more refined understanding of what it means to ask colleagues to undertake this work.

The pandemic and Weber also help us with realising that emotional life and its phenomenology are much more complex than we as individuals believe them to be, and as philosophers sometimes portray. There is likely no formula that can determine the right action, where choices can be weighed, and where guilt, shame, and reproach are negated or erased. It is likely to be impossible for anyone to be wholly virtuous, to be morally pure, and to act as such in challenging, existentially important, situations.

Tessman, in her excellent analysis of Hurricane Katrina in *When Doing the Right Thing is Impossible*,^40^ writes:

It's very distressing to think that, due to something completely outside of your own control, you might be caught in a situation in which you’re inevitably going to have to commit a moral wrongdoing. Perhaps we like to think that we can control how morally good or bad we are. If there are dilemmas, then even if we always try to do the right thing, we might end up with no right thing that we can do …. We can expect our moral lives to be less clean than we might have previously imagined because we might fail in ways that we never would have, if only it were always in our control to avoid moral failure …. The point of recognizing the phenomenon of unavoidable moral failure isn’t to identify more things that people can blamed for. Instead, the main point is to acknowledge how difficult moral life can be.

Tessman's closing line here is crucial – it is not about what clinicians can and should be blamed for, but about recognising the difficulty, and necessity, of an engaged moral life. The temptations are asceticism and quietism – withdrawal from a world to limit engagement, and to keep our moral life unstained, but at the cost of passivity and not doing any good. With Weber, we close by thinking of our politicians, our acute clinicians, and mental health practitioners, and what the future may hold for them in their reflections on the complexity of an engaged moral life over the years to come:

I would like to see what we have ‘become’, in the inner sense of the word […]. When this night slowly begins to recede, how many will still be alive of all those for whom the spring had seemed to bloom so gloriously? And what will have become of you all inwardly? Will everyone have become embittered or philistine, will they settle for a simple, dull acceptance of the world and their profession, or, and this is the third and not the most unlikely possibility: will they attempt a mystical escape from the world if they have the talent for it […]? (p. 93).^3^

## Notes

a. Participants were demographically diverse, and the minimum age was 18. In Survey 1, 55 nationalities and 51 countries were represented, and ages ranged from 18 to 90. In Survey 2, participants ranging in age from 21 to 88, from 31 different countries, participated. To collect geographically diverse reports, the team targeted UK, Japan, and Mexico residents ([^11^]). In Survey 1, 2543 responses were received and in Survey 2, 562 responses were received. Both surveys contained 42 questions. Survey 1 and Survey 2 responses were translated into English by professional translators, and the original language responses are also included in the corpus. The team articles (Froese et.al, 2021; James et.al, 2022) provide more details.

## References

[1] Runciman D . Confronting Leviathan. London: Profile Books, 2021.

[2] Froese T Broome M Carel H , et al. The pandemic experience: a corpus of subjective reports on life during the first wave of COVID-19 in the UK, Japan, and Mexico. Front Public Health 2021; 9. 10.3389/fpubh.2021.725506 PMC8418530 34490201

[3] Weber M . *The Vocation Lectures* Hackett Publishing: Indiana. Translated by Rodney Strong. 2004.

[4] Coady CAJ . The Problem of Dirty Hands. In: Zalta EN (ed.) The Stanford Encyclopedia of Philosophy 2018. Fall 2018 Edition, URL = <https://plato.stanford.edu/archives/fall2018/entries/dirty-hands/>.

[5] Moutier CY Myers MF Feist JB , et al. Preventing clinician suicide: a call to action during the COVID-19 pandemic and beyond. Acad Med 2021; 96: 624–628. 33570850 10.1097/ACM.0000000000003972

[6] Williamson V Stevelink S Greenberg N . Occupational moral injury and mental health: systematic review and meta-analysis. Br J Psychiatry 2018; 212: 339–346. 29786495 10.1192/bjp.2018.55

[7] Wiinikka-Lydon J . Mapping moral injury: comparing discourses of moral harm. J Med Philos 2019; 44: 175–191. 30877779 10.1093/jmp/jhy042

[8] Wiinikka-Lydon J . Critiquing the subject of moral injury. J Mil Ethics 2022; 21: 39–55.

[9] Awan S Diwan MN Aamir A , et al. Suicide in healthcare workers: determinants, challenges, and the impact of COVID-19. Front Psychiatry 2021; 12. 10.3389/fpsyt.2021.792925 PMC8850721 35185638

[10] Bismark M Smallwood N Jain R , et al. Thoughts of suicide or self-harm among healthcare workers during the COVID-19 pandemic: qualitative analysis of open-ended survey responses. BJPsych Open 2022; 8: e113. 10.1192/bjo.2022.509 PMC9203357 35699151

[11] Gardiner E Baumgart A Tong A , et al. Perspectives of patients, family members, health professionals and the public on the impact of COVID-19 on mental health. J Mental Health 2022: 1–10. 10.1080/09638237.2021.2022637 34983279

[12] Gilleen J Santaolalla A Valdearenas L , et al. Impact of the COVID-19 pandemic on the mental health and well-being of UK healthcare workers. BJPsych Open 2021; 7: 88. 10.1192/bjo.2021.42 PMC8082128 33910674

[13] San Juan NV Aceituno D Djellouli N , et al. Mental health and well-being of healthcare workers during the COVID-19 pandemic in the UK: contrasting guidelines with experiences in practice. BJPsych Open 2021; 7: 15. 10.1192/bjo.2020.148 PMC7844154 33298229

[14] Gray M Monti K Katz C , et al. A “mental health PPE” model of proactive mental health support for frontline health care workers during the COVID-19 pandemic. Psychiatry Res 2021; 299: 113878. 33756208 10.1016/j.psychres.2021.113878 PMC7960034

[15] Murray H Ehlers A . Cognitive therapy for moral injury in post-traumatic stress disorder. Cognit Behav Ther 2021; 14. 10.1017/S1754470X21000040 PMC7853755 34191944

[16] Williamson V Murphy D Phelps A , et al. Moral injury: the effect on mental health and implications for treatment. Lancet Psychiatry 2021; 8: 453–455. 33743196 10.1016/S2215-0366(21)00113-9

[17] Braun V Clarke V . Thematic analysis. In: Cooper H Camic PM Long DL Panter AT Rindskopf D Sher KJ (eds) APA Handbook of research methods in psychology, vol. 2. Research designs: quantitative, qualitative, neuropsychological, and biological. American Psychological Association, 2012, pp. 57–71. 10.1037/13620-004

[18] Howard R Kirkley C Baylis N . Personal resilience in psychiatrists: systematic review. BJPsych Bull 2019; 43: 209–215. 10.1192/bjb.2019.12 PMC12402926 30855001

[19] McCormack HM MacIntyre TE O’Shea D , et al. The prevalence and cause(s) of burnout among applied psychologists: a systematic review. Front Psychol 2018; 9: 1897. 30386275 10.3389/fpsyg.2018.01897 PMC6198075

[20] Gerada C . Beneath the White Coat: Doctors, Their Minds and Mental Health. Routledge, 2020.

[21] Rupert PA Kent JS . Gender and work setting differences in career-sustaining behaviors and burnout among professional psychologists. Prof Psychol: Res Pract 2007; 38: 88–96.

[22] Armstrong N . What leads to innovation in mental healthcare? Reflections on clinical expertise in a bureaucratic age. BJPsych Bull 2018; 42: 184–187. 30229718 10.1192/bjb.2018.14 PMC6189986

[23] Beale C . Magical thinking and moral injury: exclusion culture in psychiatry. BJPsych Bull 2021: 1–4. 10.1192/bjb.2021.86 PMC8914811 34517935

[24] O’Connor K Muller Neff D Pitman S . Burnout in mental health professionals: a systematic review and meta-analysis of prevalence and determinants. Eur Psychiatry 2018; 53: 74–99. 29957371 10.1016/j.eurpsy.2018.06.003

[25] Szanto T Slaby J . Political emotions. In: The Routledge handbook of phenomenology of emotion. Routledge, 2020, pp.478–494.

[26] Freudenberger HJ . Staff burn-out. J Soc Issues 1974; 30: 159–165.

[27] Maslach C Schaufeli WB Leiter MP . Job burnout. Annu Rev Psychol 2001; 52: 397–422. 11148311 10.1146/annurev.psych.52.1.397

[28] Aronsson G Theorell T Grape T , et al. A systematic review including meta-analysis of work environment and burnout symptoms. BMC Public Health 2017; 17: 64. 28302088 10.1186/s12889-017-4153-7 PMC5356239

[29] Kristensen TS Borritz M Villadsen E , et al. The Copenhagen burnout inventory: a new tool for the assessment of burnout. Work & Stress 2005; 19: 192–207.

[30] Figley C. R. (1995). *Compassion fatigue: Toward a new understanding of the costs of caring* *.*

[31] Greene T Harju-Seppänen J Adeniji M , et al. Predictors and rates of PTSD, depression and anxiety in UK frontline health and social care workers during COVID-19. Eur J Psychotraumatol 2021; 12: 1882781. 33968317 10.1080/20008198.2021.1882781 PMC8075082

[32] Ratcliffe M Ruddell M Smith B . What is a “sense of foreshortened future?” A phenomenological study of trauma, trust, and time. Front Psychol 2014: 1026. 25278917 10.3389/fpsyg.2014.01026 PMC4166378

[33] Richardson L Ratcliffe M Millar B , et al. The COVID-19 pandemic and the bounds of grief. Think 2021; 20: 89–101.

[34] Wilde L . Background feelings of belonging and psychological trauma. Psychopathology 2022; 55: 190–200. 34515210 10.1159/000518327

[35] Engebretsen KM Bjorbækmo WS . Burned out or “just” depressed? An existential phenomenological exploration of burnout. J Eval Clin Pract 2020; 26: 439–446. 31512347 10.1111/jep.13288

[36] Gustafsson G Norberg A Strandberg G . Meanings of becoming and being burnout – phenomenological-hermeneutic interpretation of female healthcare personnel’s narratives. Scand J Caring Sci 2008; 22: 520–528. 19000092 10.1111/j.1471-6712.2007.00559.x

[37] Jack K . The meaning of compassion fatigue to student nurses: an interpretive phenomenological study. J Compassionate Health Care 2017; 4: 2.

[38] Ledingham MD Standen P Skinner C , et al. “I should have known” the perceptual barriers faced by mental health practitioners in recognising and responding to their own burnout symptoms. Asia Pac J Couns Psychother 2019; 10: 125–145.

[39] Leone SS Wessely S Huibers MJH , et al. Two sides of the same coin? On the history and phenomenology of chronic fatigue and burnout. Psychol Health 2011; 26: 449–464. 20437294 10.1080/08870440903494191

[40] Tessman L . When Doing the Right Thing is Impossible. Oxford.: Oxford University Press, 2018.

